# Novel evidence that pituitary gonadotropins directly stimulate human leukemic cells-studies of myeloid cell lines and primary patient AML and CML cells

**DOI:** 10.18632/oncotarget.6698

**Published:** 2015-12-20

**Authors:** Ahmed Abdelbaset-Ismail, Sylwia Borkowska, Anna Janowska-Wieczorek, Torsten Tonn, Cesar Rodriguez, Marcin Moniuszko, Lukasz Bolkun, Janusz Koloczko, Andrzej Eljaszewicz, Janina Ratajczak, Mariusz Z. Ratajczak, Magda Kucia

**Affiliations:** ^1^ Stem Cell Institute at James Graham Brown Cancer Center, University of Louisville, KY, USA; ^2^ Pomeranian Medical University, Szczecin, Poland; ^3^ Department of Hematology, University of Alberta, Edmonton, Canada; ^4^ Transfusion Medicine, Medical Faculty Carl Gustav Carus - Technische Universtität Dresden, German Red Cross Blood Donation Service North East, Dresden, Germany; ^5^ Department of Regenerative Medicine and Immune Regulation, Medical University of Bialystok, Bialystok, Poland; ^6^ Department of Regenerative Medicine Medical University of Warsaw, Warsaw, Poland; ^7^ Department of Hematology, Medical University of Bialystok, Bialystok, Poland

**Keywords:** leukemia, lymphoma, germ line, FSH, LH

## Abstract

We recently reported that normal hematopoietic stem cells express functional pituitary sex hormone (SexH) receptors. Here we report for the first time that pituitary-secreted gonadotrophins stimulate migration, adhesion, and proliferation of several human myeloid and lymphoid leukemia cell lines. Similar effects were observed after stimulation of human leukemic cell lines by gonadal SexHs. This effect seems to be direct, as the SexH receptors expressed by leukemic cells responded to stimulation by phosphorylation of MAPK_p42/44_ and AKT_ser473_. Furthermore, in parallel studies we confirmed that human primary patient-derived AML and CML blasts also express several functional SexH receptors. These results shed more light on the potential role of SexHs in leukemogenesis and, in addition, provide further evidence suggesting a developmental link between hematopoiesis and the germline.

## INTRODUCTION

Human hematopoietic stem/progenitor cells (HSPCs) respond to several cytokines, growth factors, chemokines, and bioactive phosphosphingolipids and express the corresponding receptors for these ligands [1–4]. However, evidence has accumulated that murine and human hematopoiesis is also regulated by sex hormones (SexHs). While the role of gonadal hormones, including androgens, estrogens, and progesterone (PRG), has been demonstrated to play a role in the development of normal HSPCs [5–7], we recently reported that normal HSPCs also express functional pituitary SexH receptors for follicle-stimulating hormone (FSH), luteinizing hormone (LH), and prolactin (PRL). Specifically, injection of SexHs into mice stimulated *in vivo* expansion of HSPCs in bone marrow (BM), and, if added with suboptimal doses of cytokines and growth factors, SexHs co-stimulated *in vitro* growth of hematopoietic progenitors from all major lineages in clonogenic assays [8].

Based on results for normal HSPCs, we became interested in the role of SexHs in human hematopoietic malignancies. Interestingly, there are sex-dependent differences between males and females in development of leukemia, lymphoma, and myeloma, as males suffer more frequently from these disorders [9]. The available literature on the potential role of SexHs in malignancies is mostly limited to the potential involvement of PRL, estrogen, and androgen [10–14]. For example, it has been reported that PRL is an oncogene in rat Nb2 lymphoma cells [15, 16], and it is an autocrine growth factor for the human T cell leukemia Jurkat cell line [17]. It was also found that human CD33^+^ blasts express the PRL receptor (PRLR), and PRL increases susceptibility of these blasts to NK cells [18]. On the other hand, estrogen receptors (ESRs) and androgen receptors (ARs) were detected in SexH binding studies in cells from AML and CML patients, as well as in some established human hematopoietic cell lines [19]. Nevertheless, the effects of estrogens on leukemic cells are somehow controversial. For example, the ESR gene promoter was found to be aberrantly hypermethylated in a majority of cases of pediatric ALL, adult ALL, adult AML, and, in particular, blast crisis CML [20–23]. On other hand disruption of ESRβ in mice causes myeloproliferative disease with lymphoid crisis [24], which suggests that estrogen signaling can control proliferation of hematopoietic cells. In support of this notion, an ESR agonist has been found to have an anti-proliferative effect on lymphoma cell growth [25, 26], and 17alpha-estradiol was reported to be toxic against Jurkat cells [27]. These latter observations may *in toto* explain the protective effect of estrogens on hematopoietic malignancies in female patients [9].

While estrogens could have some protective role in developing leukemia and lymphoma, by contrast, there is, to our knowledge, no evidence for a role of pituitary SexHs, such as FSH and LH, in human malignancies. This is important, as the FSH level increases with age as a result of gonadal dysfunction and lack of negative feedback from gonadal SexHs, and it is known that age is one of the risk factors for developing hematopoietic malignancies [28, 29].

All this together prompted us to screen human leukemia cell lines (myeloid and lymphoid) as well as leukemic AML and CML blasts isolated from patients for expression of functional pituitary and gonadal SexH receptors. We found that pituitary-secreted SexHs stimulate migration, adhesion, and proliferation of several human leukemia cell lines as well as AML and CML blasts isolated from patients. This effect seems to be direct, as the receptors for these hormones respond to stimulation by phosphorylation of intracellular pathways involved in cell proliferation. We also confirmed that established human myeloid and lymphoid leukemia cell lines and primary patient blasts also responded to stimulation by gonadal SexHs.

Our study sheds more light on the paracrine regulation of leukemic cells and indicates an important novel role of pituitary SexHs in this process.

## RESULTS

### Human myeloid and lymphoid leukemia cell lines express functional SexH receptors

**Figure 1:**
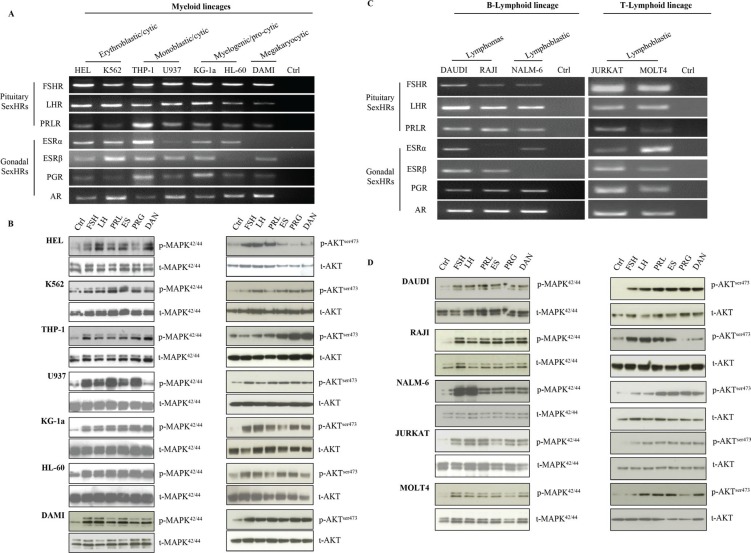
Human leukemia cell lines express functional SexH receptors Expression of SexH (pituitary and gonadal) receptors was detected in purified mRNA samples from various myeloid leukemia cell lines (**A**) as well as different B- and T-lymphoid leukemia cell lines (**C**) by reverse transcription polymerase chain reaction (RT–PCR). Samples with water only instead of cDNA were used as negative controls. Representative agarose gels of the RT-PCR amplicons obtained are shown. The effect of pituitary and gonadal SexHs on phosphorylation of p42/44 MAPK and AKT^ser473^ intracellular pathway proteins in both myeloid (**B**) and lymphoid (**D**) leukemia cell lines was investigated. These cells (2 × 10^6^ cells/mL) were starved for 5 h in RPMI containing 0.5% BSA in an incubator and afterwards stimulated for 5 min with FSH (20 IU/mL), LH (20 IU/mL), prolactin (5 μg/mL), estradiol (1 μM), progesterone (1 μM), or androgen (danazol; 4 mg/mL). The experiment was carried out twice with similar results, and representative blots are shown. FSHR, follicle-stimulating hormone receptor; LHR, luteinizing hormone/choriogonadotropin receptor; PRLR, prolactin receptor; ESRα, estrogen receptor alpha; ESRβ, estrogen receptor beta; PGR, progesterone receptor; AR, androgen receptor; SexHs, sex hormones.

Based on evidence that human normal hematopoietic cells express several SexH receptors (manuscript submitted), we became interested in whether SexH receptors are also expressed by human leukemia cells. Figure 1A–1C shows RT-PCR analysis of mRNA expression for SexH receptors in human myeloid and lymphoid cell lines, respectively. As shown in Figure 1A, we found that FSH, LH, PRL, androgen, and progesterone (PRG) receptors are expressed by all myeloid cell lines investigated in our studies: HEL, K562, THP-1, U937, KG-1a, HL-60, and DAMI. Human myeloid cell lines also express estrogen receptors α and β (ESRα and β), with the exception of DAMI cells, which express ESRβ but not ESRα. Like myeloid cell lines, the lymphoid cell lines DAUDI, RAJI, NALM-6, JURKAT, and MOLT4 express mRNA for FSH, LH, PRL, androgen, and PRG receptors (Figure 1C). At the same time, however, ESRα was not expressed by RAJI cells, and ESRβ mRNA expression was missing in the NALM-6 cell line.

To test whether these receptors are functional, we performed signal transduction assays after SexH stimulation (Figure 1B–1D). We found that stimulation of myeloid and lymphoid cells resulted in phospohorylation of MAPKp42/44 in all cell lines investigated, with the exception that androgen hormone (danazol) did not stimulate MAPKp42/44 phosphorylation in the U937 myeloid cell line (Figure 1B, left panel), despite the fact that these cells express androgen receptor (AR) mRNA. We also found that stimulation by SexHs stimulated phosphorylation of AKT at Ser473 in almost all myeloid and lymphoid cells lines (Figure 1B–1D, right panels). The only exception was the lack of phosphorylation of AKT in HEL and RAJI cells after stimulation with PRG.

### Human myeloid and lymphoid cells lines respond to SexH stimulation by enhanced migration, adhesion, and proliferation

**Figure 2:**
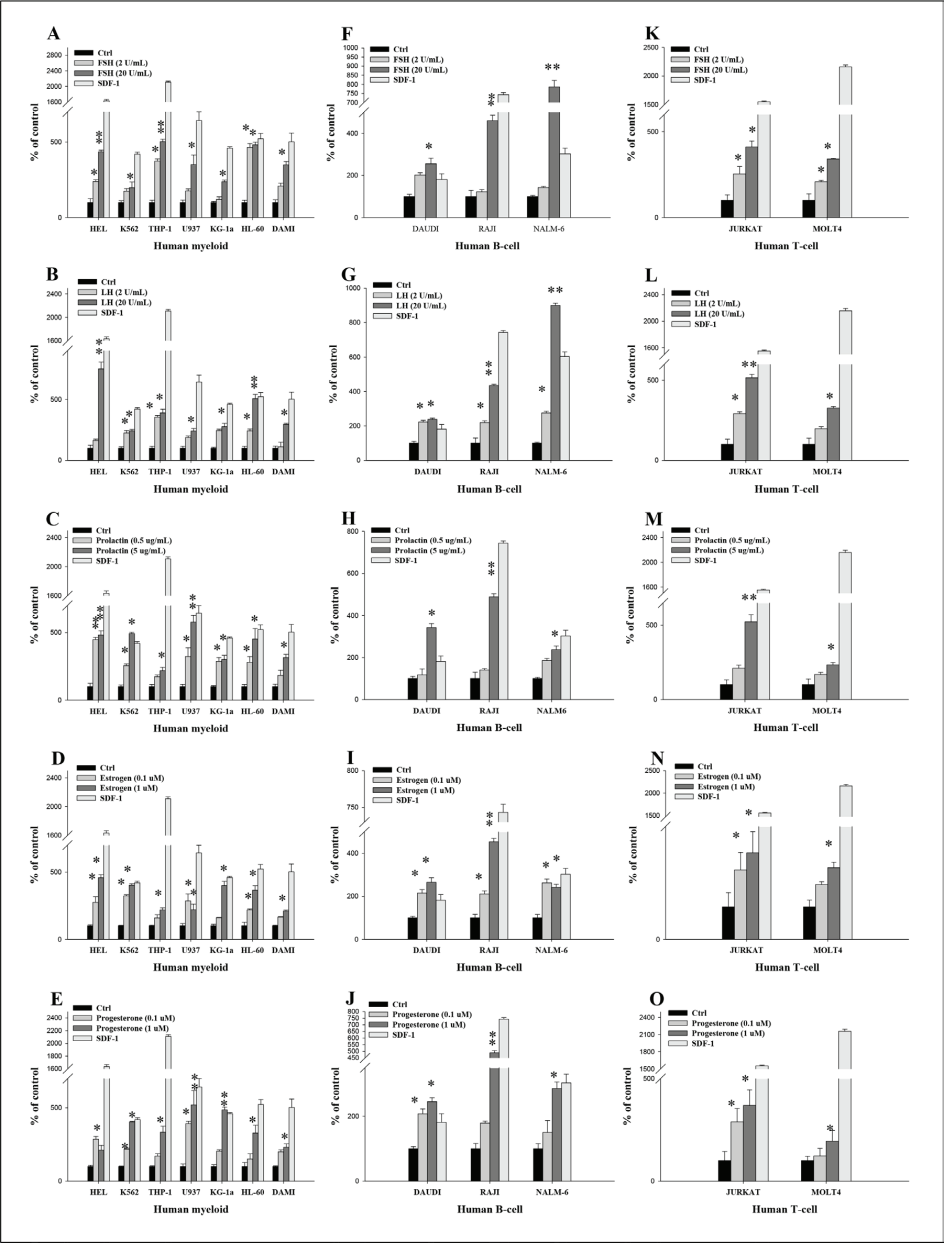
Pituitary and gonadal sex hormones are chemoattractants for human leukemia cells (**A**–**E**) Transmigration of myeloid leukemia cell lines through Transwell membranes in response to FSH (2–20 IU/mL), LH (2–20 IU/mL), prolactin (0.5–5 μg/mL), estradiol (0.1–1 μM), and progesterone (0.1–1 μM). A similar experiment was also performed to assess the effect of these hormones on migration of different B-lymphoid (**F**–**J**) and T-lymphoid (**K**–**O**) leukemic lineages. These cells were rendered quiescent in 0.5% BSA in RPMI 1640 medium for 5 h at 37°C. The effects of all these hormones on migration of all leukemia cell lines employed (10^5^ cells/100 μL/insert) were also evaluated for migration in response to stromal-derived factor 1 (300 ng/mL) and RPMI medium containing 0.5% BSA as a positive and negative control, respectively. The migrated cells were harvested and counted by FACS analysis. The negative control values are normalized to 100%. Data are displayed as means ± SD, with a statistical significance compared with the control of *p ≤ .05 and **p ≤ .01.

**Figure 3:**
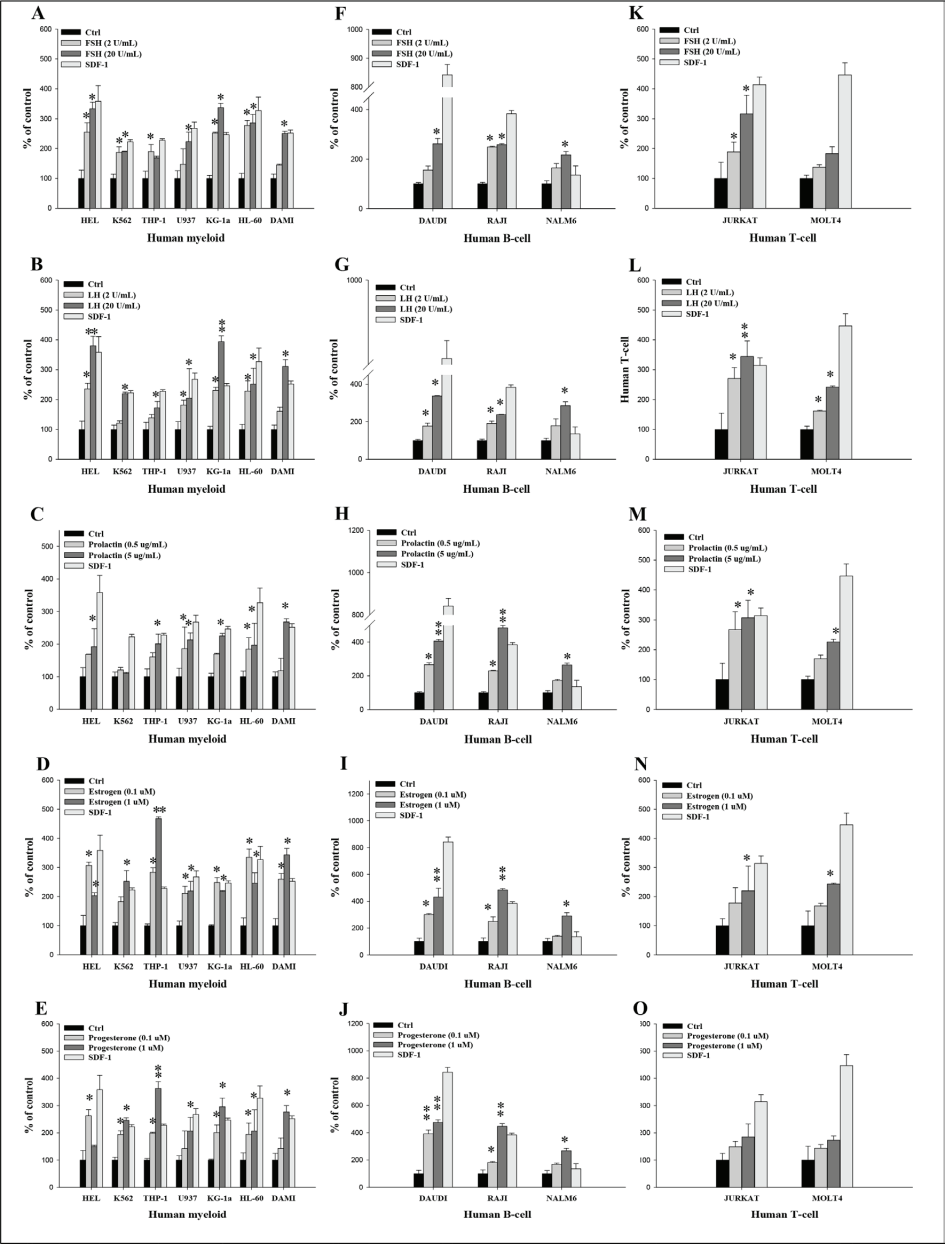
Pituitary and gonadal sex hormones enhance the adhesiveness of human leukemia cells to fibronectin (**A**–**E**) Adhesion of myeloid leukemia cell lines to fibronectin-coated surfaces in response to FSH (2–20 IU/mL), LH (2–20 IU/mL), prolactin (0.5–5 μg/mL), estradiol (0.1–1 μM), and progesterone (0.1–1 μM). A similar experiment was also performed to assess the effect of these hormones on the adhesion of different B-lymphoid (**F**–**J**) and T-lymphoid (**K**–**O**) leukemic lineages. Quiescent cells (3000 cells/100 μL) were stimulated with these hormones at the indicated concentrations in medium with 0.5% BSA for 5 min incubation at 37°C. After the non-adherent cells were removed via three consecutive washes, the number of adherent cells was measured by microscopic analysis. The effects of all these hormones on adhesion of all employed leukemia cell lines were also evaluated for adhesion versus stromal-derived factor 1 (300 ng/mL) and RPMI medium containing 0.5% BSA as a positive and negative control, respectively. The negative control values are normalized to 100%. Data are displayed as means ± SD, with a statistical significance *p ≤ .05 and **p ≤ .01 versus unstimulated control cells.

**Figure 4:**
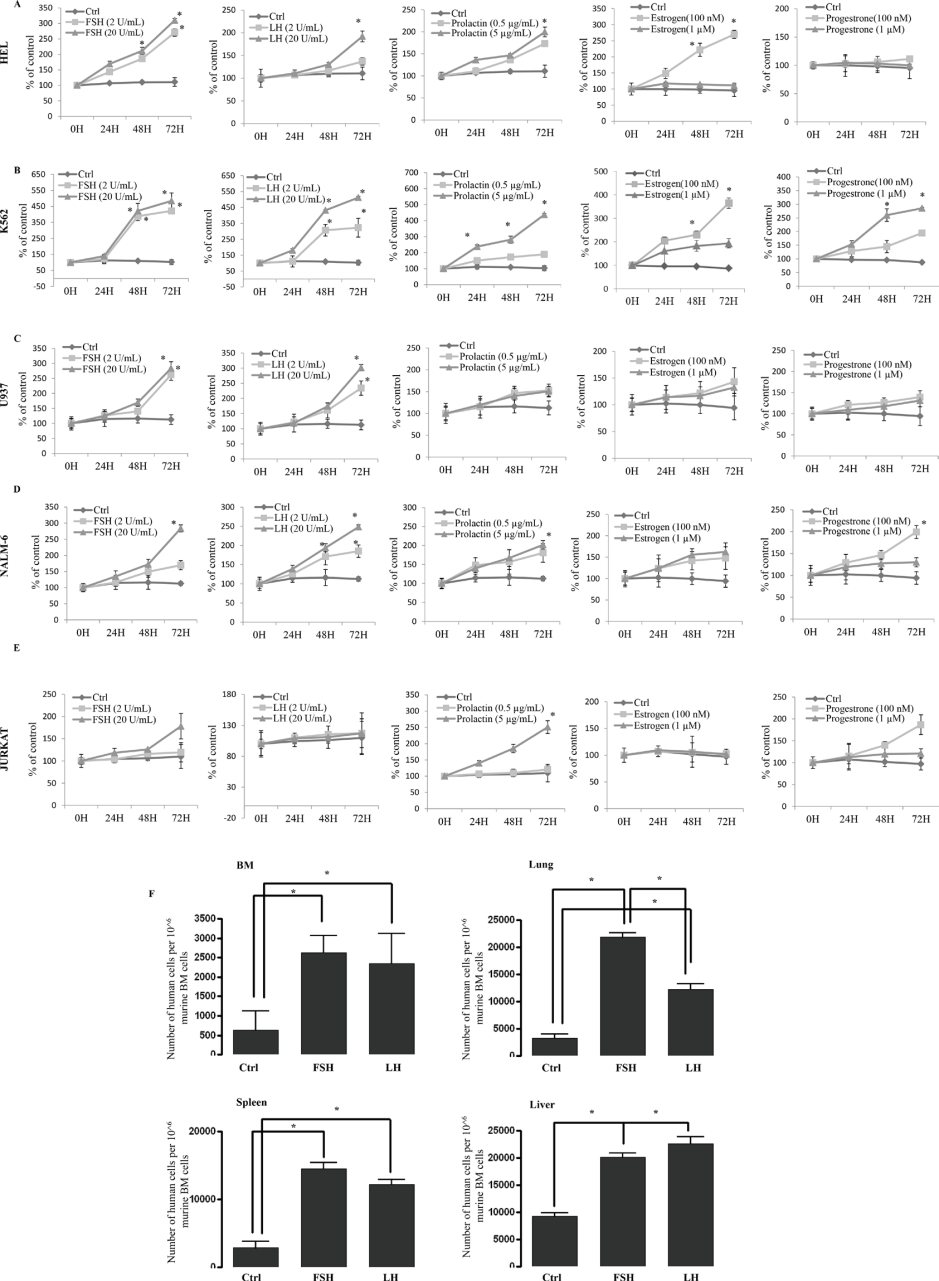
Human myeloid and lymphoid leukemia cells proliferate *in vitro* and migrate *in vivo* in response to SexHs (**A**–**C**) Proliferation of human myeloid (erythroblastic [HEL, A], erythrocytic [K562, B] and monoblastic/cytic [U937, C] leukemia cells stimulated by pituitary and gonadal SexHs in a dose-dependent manner. (**D**–**E**) Proliferation of human B-lymphoid (lymphoblastic [NALM-6, D]) and T-lymphoid (lymphoblastic [JURKAT, E]) leukemia cells by pituitary and gonadal SexHs, in a dose-dependent manner. All proliferation experiments were done in RPMI culture medium containing 0.5% BSA for 72 hours using 0.3 × 10^4^ cells/well in a 96-well plate. The negative control values are normalized to 100%. For each cell line, the experiment was repeated twice in triplicate with similar results. (**F**) Detection of *in vivo*-transplanted FSH- or LH-treated human myeloid THP-1 leukemic cells in the organs of irradiated mice post transplantation. As shown, the number of FSH- or LH-treated leukemic cells was significantly higher in isolated organs from mice than in *ex vivo*-untreated leukemic cells. Detection was performed by employing RT–qPCR for the presence of human Alu sequences in purified genomic DNA samples. For statistical comparisons, a one-way analysis of variance and a Tukey's test for post hoc analysis was carried out, and means ± SD are shown. Significance levels, *p ≤ .05 versus control.

Next, we asked whether SexHs enhance migration (Figure 2), adhesion to fibronectin (Figure 3), and proliferation (Figure 4 and Supplementary Figure 1) of human leukemic cell lines. Figure 2A–2E shows that all myeloid cells lines responded to SexHs by chemotaxis, although less than to 300 ng/ml of SDF-1. We found a similar responsiveness of B lymphoid cell lines (Figure 2F–2J) and T lymphoid cells (Figure 2K–2O). Interestingly, while all SexHs were slightly more effective than SDF-1 in the case of DAUDI cells (Figure 2F–2J), NALM6 responded better to FSH and LH than to SDF-1 (Figure 2F–2G).

In parallel adhesion studies, we found that all myeloid cell lines (Figure 3A–3E) and B cell lymphoid cell lines (Figure 3F–3J) responded to SexHs by adhesion to fibronectin at a level comparable to SDF-1. Similarly, human lymphoid T cell lines responded to stimulation by all SexHs (Figure 3K–3O), with the exception of the MOLT4 cell line, which did not respond significantly to stimulation by FSH (Figure 3K), and both JURKAT and MOLT4 cell lines, which responded weakly to PRG (Figure 3O).

Our proliferation studies performed in 0.5% BSA with or without supplementation with SexHs revealed that most of the leukemic cell lines employed in our study responded to stimulation by SexHs (Figure 4A–4E and Supplementary Figure 1A–1D). The highest responsiveness was observed to stimulation by pituitary SexHs: FSH, LH, and PRL. A somewhat weaker response was observed after stimulation with PRG and estradiol.

### Short exposure of leukemic cells with FSH and LH enhances their seeding efficiency *in vivo*

Based on the observation that FSH and LH enhance migration and adhesion of human leukemic cell lines, we exposed myeloid THP-1 cells to FSH and LH for 2 hours, injected these cells into immunodeficient beige/SCID mice (Figure 4F), and found that human leukemic cells stimulated by both pituitary SexHs had higher *in vivo* seeding efficiency to murine bone marrow, spleen, lung, and liver.

### Expression of SexH receptors by human patient myeloid leukemic cells

**Figure 5:**
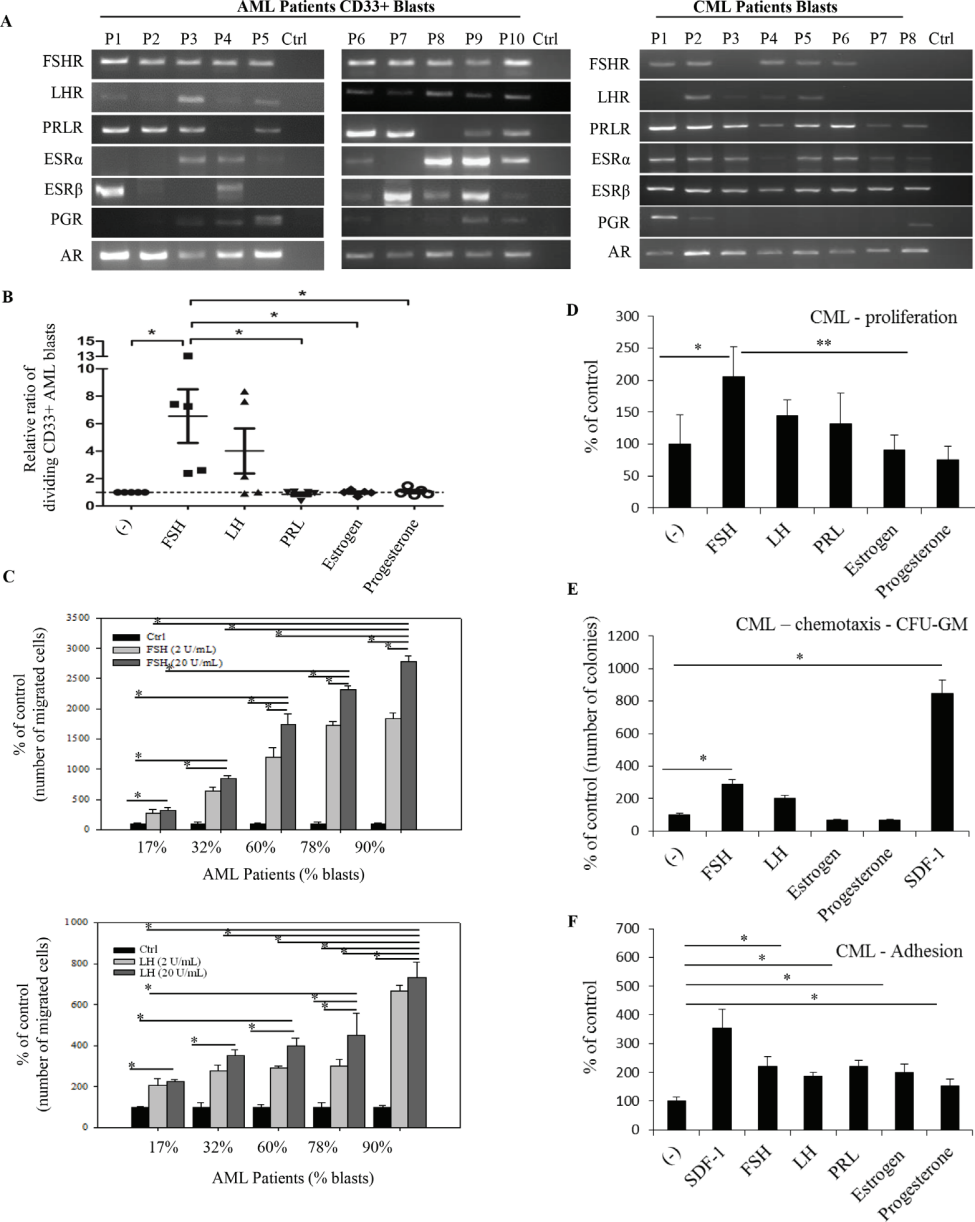
Functional SexH receptors are expressed by CD33^+^ AML and CML primary human patient blasts (**A**) Expression of SexH (pituitary and gonadal) receptor transcripts was detected in purified mRNA samples from CD33^+^ blasts sorted from AML human patients (*n* = 10) and CML human patient blasts (*n* = 8) by reverse transcription polymerase chain reaction (RT-PCR). Samples with water only instead of cDNA were used as negative controls. Representative agarose gels of the RT-PCR amplicons obtained are shown. (**B**) The effect of pituitary and gonadal SexHs on proliferation of CD33+AML blasts. The ratio of the number of dividing CD33+ AML blasts (that diluted CFSE) following stimulation with SexHs is normalized to unstimulated spontaneous blast division (dotted line). Repeated measures ANOVA test with post hoc Bonferroni multiple comparison test was used. Lines represent mean values, while whiskers represent standard error of the mean (SEM), and *p ≤ .05 is considered significant. (**C**) The effect of pituitary SexHs (FSH and LH) on transmigration of other primary mononuclear cells isolated from peripheral blood of AML patients (*n* = 8) with variable blast percentages. (**D**) Proliferation of peripheral blood mononuclear leukemia cells isolated from CML patients in response to SexHs. *P ≤ .04 and **p ≤ .03 are considered significant. (**E**) CFU-GM activity of leukemic cells isolated from primary CML patients that transmigrated through Transwell membranes in response to pituitary and gonadal SexHs. *p ≤ .05 versus control. (**F**) Pituitary and gonadal hormones stimulate the fibronectin adhesiveness of leukemia cells isolated from CML patients. These cells (2.5 × 10^3^/100 μL/well) were made quiescent and afterwards stimulated with SexHs at the indicated concentrations in RPMI medium plus 0.5% BSA for 5 min incubation. After the non-adherent cells were removed by washing three times with PBS, the number of adherent cells was then scored. The negative control values are normalized to 100%. Data from two separate experiments are merged together (each experiment has been performed in duplicate) and means ± SD are shown. *p ≤ .05 versus the control is considered significant. Stromal-derived factor 1 and 0.5% BSA in RPMI medium were used as a positive and a negative control, respectively.

Finally, to shed more light on the role of SexHs in human leukemia, we focused on primary patient cells. Figure 5A shows mRNA expression data for SexH receptors expression in purified human AML and CML blasts. We found that the FSH receptor was expressed by all 10 AML blast samples and 5 out of 8 CML blasts. At the same time, mRNA for the LH receptor was detectable by all AML and 4 out of 8 CML patient-derived cells. The PRL receptor was expressed in 8 out of 10 blast samples from AML patients and in all 8 CML blast samples. Furthermore, while AR was expressed in all AML and CML blast samples, 7 out of 10 AML and 7 out of 8 CML samples expressed ESRα, and 8 out of 10 AML and all CML blasts samples expressed ESRβ. Finally, PGR was expressed in half of AML and 3 out of 8 CML blast samples.

In proliferation assays we found that AML blasts responded by proliferation to FSH and LH exposure (Figure 5B). At the same time, PRL, estradiol, and PRG were ineffective. Similarly, we found that cells from AML patients responded in a chemotaxis assay to FSH and LH, and an increase in chemotaxis correlated with the percentage of blasts in each of the patient peripheral blood samples (Figure 5C).

In parallel experiments we observed a positive effect of FSH and, to a lesser degree, LH and PRL on proliferation of CML blasts (Figure 5D). At the same time, FSH and, somewhat more weakly, LH chemoattracted CML blasts in a Transwell migration assay (Figure 5E), and all of the SexHs evaluated in this study enhanced adhesion of CML blasts to fibronectin (Figure 5F). Thus, these results support the conclusion that SexH receptors in patient blasts, as in established leukemia cell lines, are biologically active.

## DISCUSSION

The salient observation of our work is that human leukemia and lymphoma cell lines, as well as blasts purified from primary AML and CML patient peripheral blood, express several functional SexH receptors. In particular, we demonstrate here for the first time that FSH and LH stimulate proliferation, adhesion, and chemotaxis of malignant human hematopoietic cells. Our results also provide further support for a potential developmental link between hematopoiesis and the germline.

These results are significant because of two important aspects. First, we provide further evidence that gonadal SexHs and, what is even more intriguing, pituitary SexHs may be involved in hematopoietic malignancies. To address this possibility, we performed extensive studies on several malignant hematopoietic and lymphopoietic cell lines to demonstrate that these receptors are not only expressed but, more importantly, are also functional in these cells. In all of the assays employed in this work, SexHs exerted a positive effect on proliferation, adhesion, and chemotaxis for most of the cell lines studied. However, in contrast to some other reports [33], we did not observe inhibitory effects of estrogen on proliferation or adhesion of established hematopoietic cell lines, and, more importantly, estrogen did not affect proliferation of human AML and CML blast cells. By contrast, estrogen even slightly increased adhesion of CML blasts to fibronectin. Thus, the proposed anti-leukemic therapy with estrogens [33] requires further appraisal.

In our work we also confirmed expression of PGR [34] in several human leukemic cell lines as well as human primary patient-derived AML and CML blasts. However, for the first time, we provide extensive evidence that these receptors are functional and that PRG stimulates proliferation, adhesion, and chemotaxis.

In our experiments we particularly focused on the role of pituitary SexHs. We confirmed here the stimulatory effect of PRL on human leukemic cell lines [14, 15, 17, 18, 35]. However, we made the novel observation that, in addition to PRL, FSH and LH are also potential mitogens for human leukemic cells, as we reported for normal cells [8, 36, 37]. We hypothesize that the role of FSH in proliferation of malignant hematopoietic cells requires more attention, since the FSH level increases with age, which correlates with the increase in incidence of leukemia and lymphomas in older patients [28, 29]. Moreover, based on our results, SexH therapy, and, in particular, FSH treatment should be well monitored in young patients treated with FSH after hematopoietic transplants because of transplant-conditioning-related gonadal dysfunction [38, 39].

Our results are also relevant to the biology of normal and malignant hematopoietic stem cells. Specifically, in contrast to the malignant hematopoietic cells evaluated here, normal HSPCs do not show chemotaxis in response to SexHs (manuscript submitted). This differential responsiveness of normal HSPCs versus AML and CML blasts could perhaps be explored in the clinic to more efficiently remove malignant blasts, for example, from BM cells harvested for auto-transplantation by employing migration-based *ex vivo* purging strategies.

Second, an important implication of our current study is that the expression of SexHs by normal hematopoietic stem/progenitor cells [8], and vice versa, expression of the erythropoietin receptor by germline-derived cells [40] raises an additional challenging question about the developmental origin of hematopoietic cells from a population of migrating primordial germ cells (PGC) [41–47]. Evidence has accumulated in support of this concept. First, hematopoietic cells, including leukemia cells, like germline cells, express Sall4 and respond to stimulation by bone morphogenetic protein 4 (BMP-4) [48, 49]. Second, human leukemic cells express several so-called cancer testis antigens that are detectable in germline-derived cells [50]. Third, several papers have described the sharing of chromosomal aberrations between germline tumors and leukemias or lymphomas, which suggests their common clonal origin [51, 52]. Finally, in our previous work we demonstrated that very small embryonic-like stem cells, which share several markers with migrating PGCs and express SexH receptors, can become specified into HSPCs [8, 45, 53–55]. Whether some leukemias could originate in these developmentally primitive cells was recently hypothesized by another group, but requires further study [56].

In conclusion, our results show for the first time that pituitary-secreted gonadotrophins stimulate migration, adhesion, and proliferation of established human leukemic cell lines and may stimulate primary patient-derived blasts. This effect seems to be direct, as the receptors for these hormones respond to stimulation by phosphorylation of intracellular pathways involved in cell proliferation and migration. Established human myeloid leukemia cell lines also responded to stimulation by several gonadal SexHs. Finally, our studies provide further evidence supporting a developmental link between hematopoiesis and the germline.

## MATERIALS AND METHODS

### Cell lines

Twelve human leukemia cell lines of different lineages (ATCC) were used; myeloid (erythroblastic/-cytic [HEL and K562], monoblastic/-cytic [THP-1 and U937], myelogenous/pro-myelocytic [KG-1a and HL-60], and megakaryocytic [DAMI]), B-lymphoid (lymphomas [DAUDI and RAJI] and lymphoblastic [NALM-6]), and T-lymphoblastic (JURKAT and MOLT4) cell lines. All these cell lines were maintained in Roswell Park Memorial Institute (RPMI) medium 1640 containing L-glutamine (GE Healthcare) and 10% heat-inactivated fetal calf serum (FBS; Seradigm), 100 units/mL penicillin, and 10 μg/mL streptomycin (Corning) and cultured in a humidified atmosphere of 5% CO_2_ at 37°C, with exchange of medium every 48 hours.

### Transwell chemotaxis assay

All leukemia cell lines employed were rendered quiescent by incubation in RPMI medium supplemented with 0.5% bovine serum albumin (BSA, Sigma-Aldrich) at 37°C and then seeded at a density of 10 × 10^4^ cells/well into the upper chambers of Transwell inserts with polycarbonate membranes and 8–μm pore size (Corning). The lower Boyden chambers received different concentrations of SexHs into RPMI medium with 0.5% BSA. The pituitary hormones were purchased from ProSpec (East Brunswick, NJ, USA), while the gonadal hormones were purchased from Sigma-Aldrich. The lower chambers containing stromal-derived factor 1 (SDF-1; PeproTech) and 0.5% BSA in RPMI 1640 medium were served as a positive and negative control, respectively. After 3 h of stimulation at 37°C incubation, the upper chambers were carefully removed, and the cells that had migrated to the lower chambers were collected and scored using FACS analysis.

### Adhesion of malignant hematopoietic cells to fibronectin

Cells were made quiescent for 5 h with 0.5% BSA in RPMI in a humidified atmosphere of 5% CO_2_ at 37°C. Next, the leukemia cells were stimulated with SexHs, SDF-1, or 0.5% BSA in RPMI 1640 medium. Cells were then added directly and allowed to adhere to the fibronectin-coated wells (3 × 10^3^ cells/well) in 96- well plates at 37°C. The wells were first coated with 70 μL of fibronectin (Sigma-Aldrich; 10 μg/mL) overnight at 4°C and blocked before the experiment with BSA for 2 h at 37°C. Following incubation of unstimulated and stimulated cells at 37°C, the plates were vigorously washed 3 times with PBS, and the adherent cells were counted under an inverted microscope.

### Signal transduction studies

Quiescent cells were stimulated with either 0.5% BSA in RPMI 1640 medium or SexHs for 5 min at 37°C and then lysed with RIPA lysis buffer supplemented with protease and phosphatase inhibitors (Santa Cruz Biotech). The extracted proteins were separated on a 4–12% SDS-PAGE gel, and transferred to a PVDF membrane. Phosphorylation of the intracellular kinase p42/44 mitogen-activated protein kinase (p42/44 MAPK) and AKT was detected by phosphospecific p42/44 MAPK (clone no. 9101) and pAKT (Ser473; clone no. 9271) rabbit polyclonal antibodies (Cell Signaling), respectively. Horseradish peroxidase (HRP)-conjugated goat anti-rabbit IgG was used as a secondary antibody (Santa Cruz Biotech). To ensure equal protein loading in all lanes, blots were stripped using stripping buffer (ThermoScientific), and then reprobed with appropriate anti-rabbit p42/44 MAPK (clone no. 9102) and anti-rabbit AKT (clone no. 9272) monoclonal antibodies (both from Cell Signaling). All membranes were treated with enhanced chemiluminescence (ECL) reagent (Amersham Life Sciences), dried, and subsequently exposed to film (Hyperfilm, Amersham Life Sciences). For band visualization, an automatic film processor supplied with fresh warm developer and fixer solutions was used.

### Cell proliferation

Cells were cultured in 96-well plates (Cell Star; Greiner Bio-One) at an initial density of 3 × 10^4^ cells/mL into 0.5% BSA in RPMI 1640 medium in the presence or absence of SexHs (FSH [2–20 IU/mL], LH [2–20 IU/mL], prolactin [0.5–5 μg/mL], estradiol [0.1–1 μM], or progesterone [0.1–1 μM]). The RPMI medium containing 0.5% BSA was used as a negative control, while full medium containing 10% FBS was treated as a positive control. The cell number was calculated directly after cell seeding (0 h) as well as 24, 48, and 72 h after addition of the stimulants. At these indicated time points, cells were harvested from the wells and counted using FACS.

### Reverse transcriptase-polymerase chain reaction (RT-PCR)

mRNA was extracted and purified from cells using the RNeasy Mini kit (Qiagen Inc.) after treatment with DNase I (Qiagen Inc.). The purified mRNA (200 ng) was afterwards reverse-transcribed into cDNA using Taqman Reverse Transcription Reagents (Applied Biosystems). Amplification of synthesized cDNA fragments was carried out using Amplitaq Gold polymerase (Applied Biosystems). The PCR conditions were: 1 cycle of 8 min at 95°C; 2 cycles of 2 min at 95°C, 1 min at 60°C, and 1 min at 72°C; 40 cycles of 30 s at 95°C, 1 min at 60°C, and 1 min at 72°C; and 1 cycle of 10 min at 72°C. The human sequence-specific primers were: hFSHR (sense, 5′-gcttctgagatctgtggaggtt-3′; antisense, 5′-ggacaaacctcagttcaatggc-3′), hLHR (sense, 5′-cagaggccgtccaagacac-3′; antisense, 5′-atgctccgggctc aatgtat-3′), hPRLR (sense, 5′-gagcttcttctcacagagcca-3′; antisense, 5′-aagttcacttcagggttcatgtgg-3′), hESRα (sense, 5′-aggtgccctactacctggag-3′; antisense, 5′-cggtcttttcgtat cccacct-3′), hESRβ (sense, 5′-aatggtgaagtgtggctccc-3′; antisense, 5′-acttggtcgaacaggctgag-3′), hPGR (sense, 5′-tcaactacctgaggccggat-3′; antisense, 5′-cagcatccagt gctctcaca-3′), hAR (sense, 5′-cgacttcaccgcacctgatg-3′; antisense, 5′-acttctgtttcccttcagcgg-3′). All primers were designed using the NCBI/Primer-Blast program, as at least one primer included an exon–intron boundary. Afterwards, all PCR products were analyzed by 2% agarose gel electrophoresis.

### Transplants of THP1 cells into immunodeficient mice

Before transplantation, acute myeloid leukemia cells were pretreated *ex vivo* with FSH (20 IU/mL), LH (20 IU/mL), or vehicle alone for 2 h. The cells were then washed and transplanted intravenously (3 × 10^6^ per mouse) into severe combined immunodeficient (SCID)-Beige inbred mice (*n* = 3 per group), which were irradiated with 750 cGy 24 h pre-transplantation. At 48 h post-transplantation, marrows, livers, spleens, and lungs were removed, and the presence of metastasized THP- 1 cells (i.e., murine–human chimerism) was assessed [30]. Briefly, gDNA was isolated using the QIAamp DNA Mini kit (Qiagen). Detection of human α-satellite and murine β-actin DNA levels was conducted using real-time PCR and the ABI Prism Fast 7500 Sequence Detection System (Applied Biosystems). A 25-μL reaction mixture containing 12.5 μL SYBR Green PCR Master Mix, 300 ng DNA template, and specific primers (5′-ACCACTCTGTGTCCTTCGTTCG-3′, forward and 5′-ACTGCGCTCTCAAAAGGAGTGT-3′, reverse primers for α-satellite DNA; and 5′-TTCAATTCCAACACTGTCCTGTCT-3′, forward and 5′ CTGTGGAGTGACTAAATGGAAACC-3′, reverse primers for β-actin DNA) were used. Real-time PCR conditions were as follows: 95°C (15 seconds); 40 cycles at 95°C (15 seconds) and 60°C (1 minute). The ΔCt values were determined, where Ct is the threshold cycle. The number of human cells present in the murine organs (the degree of chimerism) was calculated from the standard curve generated by mixing different concentrations of human cells with a constant number of murine cells in a linear manner.

### Patient samples

All patient samples have been obtained according to Institutional IRB guidance.

### Chronic myeloid cells

The samples of CML patients where actually samples obtained by therapeutic apheresis from pateints in blast crisis and cryopreserved in 10% DMSO and 40% *X-Vivo* 10 media in liquid nitrogen (representing > 99% blasts)

### Acute myeloid leukemia (AML) patients

Ten patients with newly diagnosed acute myeloid leukemia (AML) were recruited for the study. A diagnosis of AML was established based on the WHO classification system [31]. Complete blood counts and flow cytometry were performed in order to confirm the presence of blast cells. Detailed clinical, phenotypical, and molecular characteristic of recruited AML patients are presented in supplemental Table 1. EDTA-anticoagulated whole blood was obtained from these AML patients and immediately processed. Peripheral blood mononuclear cells (PB- MNCs) were isolated by means of density-gradient centrifugation using Histopaque 1077 medium (Sigma-Aldrich).

### Analysis of proliferative responses of CD33^+^ AML blasts to stimulation with sex hormones

PB-MNCs collected from AML patients (*n* = 10) were labeled with carboxyfluorescein diacetate succinimidyl ester (CFSE; Sigma-Aldrich) as previously described [32]. CFSE-stained PB-MNCs were cultured alone or in the presence of SexHs, namely FSH (20 IU/mL), LH (20 IU/mL), prolactin (5 μg/mL), estrogen (1 μM), and progesterone (1 μM). Following 96 h of culture, cells were stained with mouse anti-human anti-CD33 PE monoclonal antibodies (Becton Dickinson) and analyzed for CFSE dilution using a FACSCalibur cell analyzer (Becton Dickinson). A fluorescence minus one (FMO) control was applied for setting compensation and to assure correct gating of CD33^+^ blasts. The data obtained were analyzed using FlowJo version 7.6.5 software (TreeStar). The proliferative data for different SexHs are presented as the relative ratio of the number of dividing CD33^+^ blasts, assessed by CFSE dilution, normalized to the CFSE dilution observed in unstimulated spontaneously dividing CD33^+^ blasts.

### CD33^+^ AML blast separation and RT-PCR

CD33^+^ AML blast isolation was performed using magnetic cell sorting (MACS, Miltenyi Biotec), according to the manufacturer's instructions. Briefly, PB-MNCs obtained from AML patients were labeled with MicroBeads conjugated to mouse anti-human anti-CD33 antibodies (Militenyi Biotec) in staining buffer (autoMACS running buffer). Following removal of the column from the magnetic field, CD33^+^ AML blasts were collected. Next, mRNAs were collected from CD33^+^ AML (*n* = 10) blast patient primary cells using the RNeasy Micro kit (QIAGEN). For cDNA synthesis, 10 μg of mRNA from each sample was reverse transcribed using a high-capacity cDNA reverse-transcription kit (Life Technologies). The amplification of synthesized cDNA fragments was carried out using Amplitaq Gold polymerase as described above.

### Chemotaxis studies

Other fresh primary PB-MNCs that had been collected directly from AML leukemia patients (*n* = 8), with variable percentages indicated for their blasts, were also subjected to transmigration assays in response to FSH (2–20 IU/mL), LH (2–20 IU/mL), or medium free from these stimulants.

### Chronic myeloid leukemia patients (CML)

#### SexH receptor expression

Analysis was also carried out by RT-PCR to evaluate the expression of SexH receptors by primary leukemia cells isolated from CML (*n* = 8) blast patients. Purification of mRNA, cDNA synthesis, and amplification of synthesized cDNA fragments were fulfilled as described here.

#### Cell adhesion studies

Quiescent primary CML cells were stimulated with FSH (5 IU/mL), LH (5 IU/mL), prolactin (1 μg/mL), estradiol (100 nM), and progesterone (100 nM). Cells (2.5 × 10^4^/mL) were added to fibronectin-coated 96- well plates and incubated for 5 min at 37°C. Following incubation, the plates were washed and adherent cells were counted.

For cell migration and granulocyte/macrophage colony-forming unit (CFU-GM) assays, primary CML cells were resuspended in assay medium (RPMI medium containing 0.5% BSA). FSH (5 IU/mL), LH (5 IU/mL), estradiol (100 nM), or progesterone (100 nM) were added to the lower chambers of a Transwell plate. Next, cells were loaded onto the upper chambers, and then incubated for 3 h at 37°C and 5% CO_2_. The migrated CML cells were afterwards harvested, scored, and subjected to CFU- GM colony assay in which the following concentrations of growth factor and cytokine were respectively used: rhGM-CSF (25 ng/mL) and rhIL-3 (10 ng/mL) as previously described (both reagents were purchased from R & D Systems; Minneapolis, MN, USA) [8].

#### Proliferation assay

To evaluate proliferation efficiency [8], primary CML cells were plated with methylcellulose base medium, growth factors (GM-CSF [25 ng/mL] and IL-3 [10 ng/mL]) and hormones (FSH [5 IU/mL], LH [5 IU/mL], prolactin [1 μg/mL], estradiol [100 nM], and progesterone [100 nM]). Importantly, suboptimal doses of these factors (i.e., only 1/10 of the abovementioned concentrations) were used. After 14 days of culture at 37°C and 5% CO_2_ atmosphere, the numbers of CFU-GM colonies were scored using an inverted microscope (Olympus, Center Valley, PA, USA).

#### Data analysis

Statistical analysis was carried out using GraphPad Prism 6 (GraphPad Software Inc) and Sigma software (Sigma Software Inc). All data were presented as mean ± SD. Statistical analysis of the data was done using one-way ANOVA and Tukey's test for post hoc pairwise multiple comparison. Repeated measures ANOVA tests with post hoc Bonferroni multiple comparison tests were used to determine differences in the effects of SexHs on AML blast proliferation. In all analyses, *p* ≤ .05 and *p* ≤ .01 are considered significant, while in CML proliferation *p* ≤ .03 and *p* ≤ .04 are defined as significant.

## SUPPLEMENTARY MATERIALS FIGURES AND TABLES


