# Supplementary material for: Novel evidence that pituitary gonadotropins directly stimulate human leukemic cells-studies of myeloid cell lines and primary patient AML and CML cells
Source: Oncotarget. 2015 Dec 20;7(3):3033–46. doi: 10.18632/oncotarget.6698 (PMC4823088; doi:10.18632/oncotarget.6698)
Supplement: Supplementary file 1 [file oncotarget-07-3033-s001.pdf]

Novel evidence that pituitary gonadotropins directly stimulate human leukemic cells -studies of myeloid cell lines and primary patient AML and CML cells

Supplementary Materials

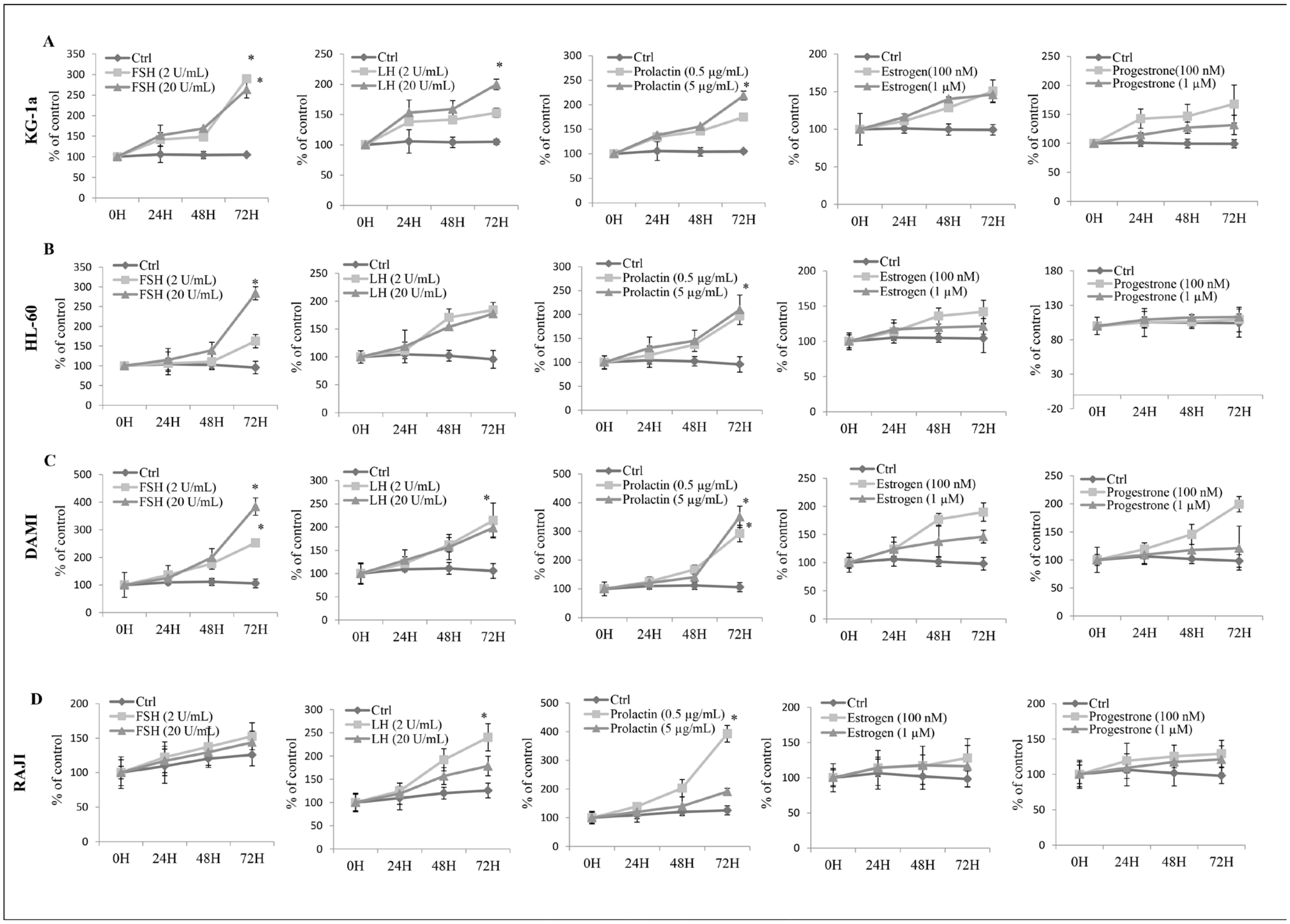

Supplementary Figure 1: Human myeloid and lymphoid leukemia cells proliferate *in vitro* and migrate *in vivo* in response to SexHs. (**A–C**) Proliferation of human myeloid (myelogenous [KG-1a, A], pro-myelocytic [HL-60, B], megakaryoblastic/cytic [DAMI, C]) leukemia cells by pituitary and gonadal SexHs in a dose-dependent manner. Panels (**D**) Proliferation of human B-lymphoid (lymphomas [RAJI, D], leukemia cells by pituitary and gonadal SexHs, in a dose-dependent manner. For statistical comparisons, a one-way analysis of variance and a Tukey's test for post hoc analysis was carried out, and means ± SD are shown. Significance levels, *$p \leq .05$ versus control.

Supplementary Table S1: Clinical, phenotypical and molecular characteristics of AML patients

Number of patients	10
Median (range) age (years)	57.5 (21–62)
Median (range) white blood cell count (G/l)	57.2 (9.9–199.5)
Median (range) of the blastic cells in peripheral blood	76 (21–97)
Median (range) of the CD33 positivity within blastic cells [%]	92 (78–98)
AML according to FAB classification	10
AML, minimally differentiated, M0	2
AML without maturation, M1	2
AML with maturation, M2	3
Acute myelomonocytic leukemia (AMMoL), M4	3
Karyotype	46XX/46XY
FLT3-ITD/NMP1$_{mut}$/*CEBPA*$_{mut}$	0/0/0

Cytogenetic and molecular analysis, including fluorescence in situ hybridization (FISH) assays (of AML1/ETO, CBFß/ MYH11, MLLT3–MLL and frequently mutated genes FLT3–ITD, NPM1, CEBPA) were performed to determine the risk group as recommended by WHO guidelines. Based on the abovementioned parameters, all included patients presented with a normal karyotype (46XX/46XY), and none of them presented with either mutated core binding factor leukemia (CEBPA$_{mut}$), mutated nucleophosmin (NPM1$_{mut}$), or internal tandem duplication of Fms-like tyrosine kinase 3 (FLT3–ITD). All patients were hospitalized in the Department of Hematology of the Medical University of Bialystok in year 2014 and were qualified for 7-day induction chemotherapy regimens corresponding to the standard therapy based on the Polish Adult Leukemia Group: cytarabine was delivered as a continuous IV infusion for 7 consecutive days at a dose of 200 mg/m^2; anthracycline was delivered for 3consecutive days as an IV push at a dose of 50 mg/m^2; and cladribine was administered for 5 days as an IV push at a dose of 5 mg/m^2 (DAC schedule) [32].

Abbreviations: AML- acute myeloid leukemia, NPM1$_{mut}$ -mutated nucleophosmin, CEBPA$_{mut}$ – mutated core binding factor leukaemia , FLT3-ITD- internal tandem duplication of Fms-like tyrosine kinase 3

Supplementary Table S2: Summary of the results obtained from experiments conducted on various human leukemia and lymphoma cell lines

		Receptor expression							Migration										Adhesion										Proliferation										MAPKp42/44					pAKTser473				
		FSHR	LHR	PRLR	ESRα	ESRβ	PGR	AR	FSH		LH		PRL		ES		PRG		FSH		LH		PRL		ES		PRG		FSH		LH		PRL		ES		PRG		FSH	LH	PRL	ES	PRG	FSH	LH	PRL	ES	PRG
									L	H	L	H	L	H	L	H	L	H	L	H	L	H	L	H	L	H	L	H	L	H	L	H	L	H	L	H	L	H										
Myeloid lineages	HEL	+	+	+	+	+	+	+	S	S*	N	S*	S*	S*	S	S	S	N	S	S	S	S*	N	S	S	S	S	N	S	S†	N	S	N	S	S†	N	N	N	M	H	M	M	L	H	H	H	M	L
	K562	+	+	+	+	+	+	+	N	S	S	S	S	S	S	S	S	S	S	S	N	S	N	N	N	S	S	S	S†	S†	S†	S†	N	S‡	S†	N	N	S†	M	M	H	H	M	M	H	M	H	H
	THP-1	+	+	+	+	+	+	+	S	S*	S	S	N	S	N	S	N	S	S	N	N	S	N	S	S	S*	S	S*	NT										H	M	M	M	M	L	M	M	H	H*
	U937	+	+	+	+	+	+	+	N	S	N	S	S	S*	S	S	S	S*	N	S	S	S	S	S	S	S	N	S	S	S	S	S	N	N	N	N	N	N	H*	H	H*	H	H*	M	M	M	M	M
	KG-1a	+	+	+	+	+	+	+	N	S	N	S	S	S	N	S	N	S*	S	S	S	S*	N	S	S	S	S	S	S	S	N	S	N	S	N	N	N	N	M	M	M	H	H	H	H	H	M	H
	HL-60	+	+	+	+	–	+	+	S	S	S	S*	S	S	S	S	N	S	S	S	S	S	S	S	S	S	S	S	N	S	N	N	N	S	N	N	N	N	M	M	M	M	M	M	M	M	M	M
	DAMI	+	+	+	–	+	+	+	N	S	N	S	N	S	N	S	N	S	N	S	N	S	N	S	S	S	N	S	S	S	S	N	S	S	N	N	N	N	H	H	M	H	M	H	H	H	H	H
Lymphoid lineages	DAUDI	+	+	+	+	+	+	+	N	S	S	S	N	S	S	S	S	S	N	S	S	S	S	S*	S	S*	S*	S*	NT										M	M	H	M	M	M	M	H	H	H
	RAJI	+	+	+	–	+	+	+	N	S*	S	S*	N	S*	S	S*	N	S*	S	S	S	S	S	S*	S	S*	S	S*	N	N	S	N	S	N	N	N	N	N	H	H	H	H	H	H	H	M	M	L
	NALM-6	+	+	+	+	–	+	+	N	S*	S	S*	N	S	S	S	N	S	N	S	N	S	N	S	N	S	N	S	N	S	S	S†	N	S	N	N	S	N	H*	H*	H	H	H	M	M	H	H	H
	JURKAT	+	+	+	+	+	+	+	S	S	S	S*	N	S*	S	S	S	S	S	S	S	S*	S	S	N	S	N	N	N	N	N	N	N	S	N	N	N	N	H	H	H	H	M	M	M	M	M	M
	MOLT4	+	+	+	+	+	+	+	S	S	N	S	N	S	N	S	N	S	N	N	S	S	N	S	N	S	N	N	NT										H	M	M	M	M	L	M	M	M	L

There are summarized receptor expression studies and biological responses of cells to SexH stimulation (migration, adhesion, proliferation, MAPKp42/44 and AKT phospohorylation)

Abbreviations: FSH, follicle-stimulating hormone; LH, luteinizing hormone; PRL, prolactin; ES, estrogen; PRG. progesterone; L, low dose; H, high dose.

Receptor expression: +, expressed; –, not expressed.

Migration, adhesion: S*, highly significant ($p \leq .01$); S, significant ($p \leq .05$); N, non significant.

Proliferation: S‡, significant at all time points ($p \leq .05$); S†, significant only at 48H and 72H ($p \leq .05$); S, significant only at 72H ($p \leq .05$), N, non significant; NT, not tested.

Phosphorylation studies: H*, very high singling; H, high; M, moderate; L, low.
